# Young lady with a typical solid–cystic abdominal mass

**DOI:** 10.1002/ccr3.852

**Published:** 2017-02-15

**Authors:** Saket Kumar, Rahul Rahul, Abhijit Chandra

**Affiliations:** ^1^ Surgical Gastroenterology DepartmentShatabdi HospitalKing George's Medical UniversityLucknowUttar Pradesh226003India

**Keywords:** Central pancreatectomy, cystic lesion, pancreas, pseudopapillary tumor

## Abstract

Solid pseudopapillary tumor is a rare pancreatic neoplasm that typically affects young women. Characteristic CT appearance is that of a mixed‐density lesion with solid component peripherally and cystic component centrally. Even larger tumors are well encapsulated with sharp demarcation, amenable to complete resection.

## Case Question

A 25‐year‐old woman presented with dull‐aching pain in the upper abdomen for 1 year. Per‐abdominal examination revealed a fixed, firm, and nontender lump in the epigastrium. Blood counts, liver function test, and serum amylase–lipase were within normal limit. Computed tomography image and intra‐operative pictures are as below. What is the correct diagnosis?

- Gastrointestinal stromal tumor of stomach
- Hydatid cyst of pancreas
- Solid pseudopapillary tumor of pancreas
- Pseudocyst of pancreas

## Correct Answer: C

**Figure 1:**
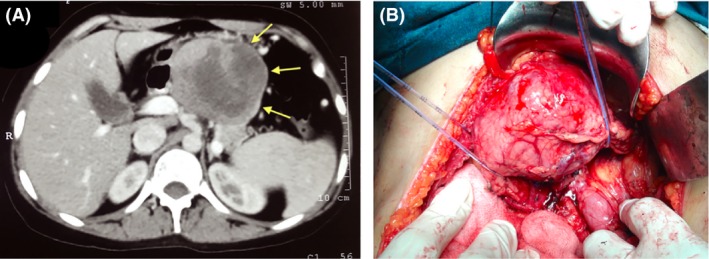
(A) Computed tomography showing a solid–cystic tumor arising from the body of pancreas. On exploration, a well‐circumscribed capsulated tumor was found arising from the body of pancreas (B).

Solid pseudopapillary tumor of pancreas is classically seen in young women and commonly present with upper abdominal pain 1. In the above case, CT of abdomen revealed a solid–cystic mass (10 × 8 × 8 cm) in the body of the pancreas (Fig. 1A). Considering the typical clinical presentation and characteristic CT findings, diagnosis of solid pseudopapillary tumor was almost certain. On exploration, a well‐circumscribed tumor arising from the body of pancreas was found. The tumor could be mobilized circumferentially with adequate proximal and distal margins (Fig. 1B). Spleen‐preserving central pancreatectomy with Roux‐en‐Y pancreaticojejunostomy was performed. Cut section of the tumor revealed a capsulated solid–cystic tumor. Histopathology was consistent with solid pseudopapillary tumor of the pancreas, and thus, final diagnosis was established. The postoperative recovery was uneventful, and she was discharged on the day 6 of surgery. She was followed up at every 3 months and CT of abdomen was performed at 6 and 12 months. She remained asymptomatic and disease‐free during the follow‐up of 1 year.

Although hydatid cyst of the pancreas has been reported, it is extremely rare. CT scan in the present case shows a mass with solid–cystic component. This does not correlate with features of hydatid cysts, where lesion is cystic with multiple daughter cysts within it.

Pseudocyst is the most common form of cystic lesion in pancreas. However, it more frequently affects men and is associated with a history of pancreatitis or trauma in majority of the cases. CT picture shows a homogenous hypodense lesion, without any solid component. Absence of these clinical and radiological features suggested different diagnosis in the above case.

Similarly, GIST most commonly arises from the stomach and presents with epigastric pain, lump, and recurrent vomiting resulting from gastric outlet obstruction. Moreover, it is diagnosed more commonly in elderly men. CT shows predominantly solid tumor, although central necrosis or hemorrhage may produce solid–cystic‐like picture. In the present case, the mass was seen arising from the pancreas, which could be completely isolated from the surrounding structures, making the diagnosis of gastric GIST unlikely.

## Conflict of Interest

None declared.

## Authorship

SK: wrote the manuscript and is the operating surgeon. RR: collected images and data, wrote the manuscript, and reviewed the literature. AC: edited the manuscript, performed critical revision, and reviewed the literature.
